# Do Colonic Mucosal Tumor Necrosis Factor Alpha Levels Play a Role in Diverticular Disease? A Systematic Review and Meta-Analysis

**DOI:** 10.3390/ijms24129934

**Published:** 2023-06-09

**Authors:** Cristina Maria Sabo, Mohamed Ismaiel, Abdulrahman Ismaiel, Daniel-Corneliu Leucuta, Stefan-Lucian Popa, Simona Grad, Dan L. Dumitrascu

**Affiliations:** 1 2nd Department of Internal Medicine, “Iuliu Hatieganu” University of Medicine and Pharmacy, 400006 Cluj-Napoca, Romania; cristina.marica90@yahoo.com (C.M.S.); popa.stefan@umfcluj.ro (S.-L.P.); costinsimona_m@yahoo.com (S.G.); ddumitrascu@umfcluj.ro (D.L.D.); 2 Department of General Surgery, Altnagelvin Hospital, Londonderry BT47 6LS, UK; dr.mohamed.sami.i@gmail.com; 3 Department of Medical Informatics and Biostatistics, “Iuliu Hatieganu” University of Medicine and Pharmacy, 400349 Cluj-Napoca, Romania; dleucuta@umfcluj.ro

**Keywords:** diverticular disease (DD), tumor necrosis factor-alpha (TNF-α), inflammatory markers, biomarkers, irritable bowel syndrome

## Abstract

Diverticular disease (DD) is the most frequent condition in the Western world that affects the colon. Although chronic mild inflammatory processes have recently been proposed as a central factor in DD, limited information is currently available regarding the role of inflammatory cytokines, such as tumor necrosis factor-alpha (TNF-α). Therefore, we conducted a systematic review and meta-analysis aiming to assess the mucosal TNF-α levels in DD. We conducted a systematic literature search using PubMed, Embase, and Scopus to identify observational studies assessing the TNF-α levels in DD. Full-text articles that satisfied our inclusion and exclusion criteria were included, and a quality assessment was performed using the Newcastle–Ottawa Scale (NOS). The principal summary outcome was the mean difference (MD). The results were reported as MD (95% confidence interval (CI)). A total of 12 articles involving 883 subjects were included in the qualitative synthesis, out of which 6 studies were included in our quantitative synthesis. We did not observe statistical significance related to the mucosal TNF-α levels in symptomatic uncomplicated diverticular disease (SUDD) vs. the controls (0.517 (95% CI −1.148–2.182)), and symptomatic vs. asymptomatic DD patients (0.657 (95% CI −0.883–2.196)). However, the TNF-α levels were found to be significantly increased in DD compared to irritable bowel disease (IBS) patients (27.368 (95% CI 23.744–30.992)), and segmental colitis associated with diverticulosis (SCAD) vs. IBS patients (25.303 (95% CI 19.823–30.784)). Between SUDD and the controls, as well as symptomatic and asymptomatic DD, there were no significant differences in the mucosal TNF-α levels. However, the TNF-α levels were considerably higher in DD and SCAD patients than IBS patients. Our findings suggest that TNF-α may play a key role in the pathogenesis of DD in specific subgroups and could potentially be a target for future therapies.

## 1. Introduction

Diverticulosis, a prevalent colonoscopic finding, is described as the presence of sac-like colonic protrusions. It is asymptomatic in most cases; however, about one-fourth will progress to diverticular disease (DD) [1]. This could be further divided into symptomatic uncomplicated diverticular disease (SUDD) and symptomatic complicated, which includes acute diverticulitis [1]. Additionally, segmental colitis associated with diverticulosis (SCAD) has been described as a type of colonic inflammatory disease with localized and non-granulomatous features typically limited to the sigmoid colon [2].

Diverticulosis is common in both Eastern and Western countries; however, the highest incidence was reported in the United States, Australia, and Western Europe [3]. There are differences in the anatomic diverticular distribution based on geographic areas. It mainly occurs at the right colon in Asian countries and the left colon in Western countries [4]. There is a lifetime risk from 10 to 25% of developing diverticulitis in those diagnosed with diverticulosis, and it is associated with substantial healthcare burden and morbidity [5]. About 12% of diverticulitis cases are complex, involving abscess, fistula, stricture, or perforation [6].

The pathophysiology of diverticulosis is poorly understood; nevertheless, numerous modifiable and non-modifiable factors have been associated with a high risk of diverticulosis development [7]. These include age, smoking, obesity, alcohol ingestion, lack of dietary fibers, genetic and environmental factors, abnormal colonic motility, microbiota imbalance, structural colonic wall aberrations, neuro-immune dysregulation, and mucosal inflammation [8,9]. The processes implicated in the transformation to a symptomatic diverticular status from an asymptomatic one are not clearly understood [9]. The prevalence of DD increases with age and is comparable between women and men [10].

Inflammation, both acute and chronic, plays a major role in DD and diverticulitis development and recurrence [11,12]; however, its role in the pathogenesis of diverticulosis is not very well understood at the moment [13]. Tumor necrosis factor alpha (TNF-α) is a key pro-inflammatory cytokine produced by lymphocytes, macrophages, and natural killer cells [14,15]. It is involved in multiple cellular processes and plays a role in the modulation of inflammatory pathways, such as inducible nitric oxide synthase and cyclooxygenase-2 [16].

Dysregulated TNF-α signaling has been associated with multiple inflammatory and autoimmune diseases, including rheumatoid arthritis, psoriasis, and inflammatory bowel disease (IBD) [14,17]. In IBD, there is an overexpression of numerous pro-inflammatory cytokines, including TNF-α, which results in tissue damage and stimulation of the immune system (innate and adaptive), causing chronic inflammation [16]. The use of immunotherapy, such as infliximab, a TNF-α monoclonal antibody in IBD, has been associated with remission in moderate to severe IBD cases, which reveals the significance of TNF-α in bowel disease pathology [18].

Additionally, several studies have reported elevated colonic mucosal TNF-α levels in symptomatic diverticulosis, including acute uncomplicated diverticulitis and SUDD, while also being associated with disease severity [17]. In contrast, other studies have shown no relationship between colonic mucosal TNF-α levels and symptomatic status in diverticulosis [19]. There are discrepancies in the literature in regard to the TNF-α levels between different DD and its association with the symptomatic status. Hence, we conducted a systematic review and meta-analysis to evaluate TNF-α expression in DD.

## 2. Methods

This systematic review and meta-analysis was written as per the preferred reporting items for systematic reviews and meta-analyses (PRISMA) 2020 statement [20].

### 2.1. Data Sources and Search Strategy

We performed a computerized search on PubMed, Embase, and Scopus electronic databases to identify observational studies evaluating TNF-α levels in DD. The utilized search string for PubMed was ((“Tumor Necrosis Factor-alpha” (Mesh)) OR (“Tumor Necrosis Factor-alpha” (All Fields))) AND ((“Diverticular Diseases” (Mesh)) OR (“Diverticular Diseases” (All Fields))), and a similar one was used according to each database. Additionally, a manual search was conducted for pertinent missed publications via screening the references of the included articles. The search included published articles from inception until 12 October 2022 by two autonomous investigators (C.M.S. and A.I.). In the event of disparities, a consensus was attained following discussion. During the search, no restrictions or filters on country, language, or duration were selected. The screening of titles and abstracts for eligibility was conducted. Subsequently, full-text articles that fulfilled our inclusion and exclusion criteria were evaluated. Data extraction was conducted by an investigator (C.M.S.) and validated by another investigator (A.I.). Differences were settled by addressing the source article. The data extracted comprised the author names, year of publication, country, study design, studied population, total sample size, mean age, sex distribution, type of DD, TNF-α levels (mean ± SD or median (IQR)), and main study outcome, which were collated and presented in the manuscript text.

### 2.2. Eligibility Criteria

The inclusion criteria for the original articles in this systematic review and meta-analysis were the following: (1) observational cohort, case-control studies, or cross-sectional evaluations of the TNF-α levels in DD; (2) colonic diverticulosis confirmed by colonoscopy, imaging, or according to each study criteria; (3) human studies with no restrictions on sex, race, or ethnicity; (4) studies published in English, French, German or Romanian languages.

The exclusion criteria were the following: editorials, letters, commentaries, conference abstracts, case reports, practice guidelines, and abstracts lacking a full article and review articles.

### 2.3. Risk of Bias Assessment in Individual Studies

A quality assessment of the included studies, appraising the internal validity and risk of bias, was conducted similarly using the Newcastle–Ottawa scale (NOS) tool for cross-sectional studies by two autonomous authors (C.M.S. and M.I.) [21,22]. In the event of disparity between the two investigators, a consensus was achieved through a discussion with a third investigator (D.C.L.). All assessed studies were scored based on how many stars were obtained. After confirming the criteria for the selection, comparability, and outcome sections, the study was given a rating that ranged from 0 to 9 stars. To assess the included studies’ quality in a quantifiable way, the total number of stars in each research was added together. Moreover, the methodological quality assessment outcomes did not impact the eligibility of the included studies. The absence of reporting on the confidence intervals in the presence of the *p*-value and descriptive statistics was not considered as necessary to remove one star since it could be computed (according to the recommendation in the Cochrane Handbook).

### 2.4. Summary Measure and Synthesis of Results

Statistical analysis was performed using R with the metafor package (OpenMeta (Analyst)) [23,24]. The mean difference (MD) of the TNF-α levels was the primary summary outcome. Q test and I² statistics were used to assess between-study heterogeneity. According to the recommendations in the Cochrane Handbook for identifying and measuring heterogeneity [25], an effect size with an I² from 0% to 40% was considered low heterogeneous, an I² from 30% to 60% was considered moderately heterogeneous, an I² from 50% to 90% was considered substantially heterogeneous, and an I² from 75% to 100% was considered as considerable heterogeneity. We used the random-effects model and MD to analyze the estimated total effect size. Combining groups in the studies with several subgroups of DD patients or control subjects without a total group was performed according to the Cochrane Handbook recommendations. Subgroup analysis was conducted comparing the TNF-α levels in SUDD patients vs. the controls, SUDD patients vs. asymptomatic patients, DD patients vs. irritable bowel syndrome (IBS) patients, and SCAD patients vs. IBS patients. The data from each study was reported as the estimated MD with 95% CI, lower bound, upper bound, standard error, and *p*-value. Statistical significance was considered when the *p*-value was <0.05. Analysis was performed when at least two studies assessed similar groups and revealed the same outcome via mean and SD, or median (IQR) of the TNF-α levels.

## 3. Results

### 3.1. General Results

**Figure 1:**
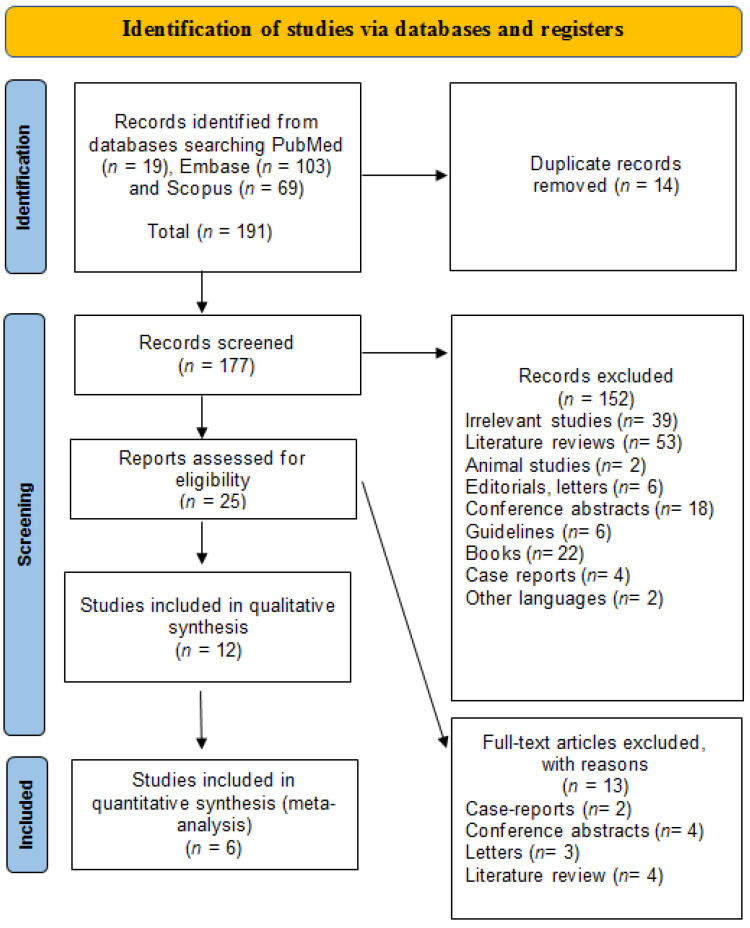
PRISMA flow diagram: identification, screening, and inclusion stages in our systematic review and meta-analysis.

Figure 1 delineates the PRISMA flow diagram displaying the performed search strategy. The preliminary search yielded 191 articles (PubMed *n* = 19, Embase *n* = 103, Scopus *n* = 69 articles). A total of 14 studies were eliminated as they were duplicates. Following the exclusion of duplicates, 177 articles were preliminary screened for the fulfillment of the inclusion and exclusion criteria by evaluating the title and abstract. Throughout the screening stage, 152 articles were excluded for the following reasons: (1) 39 were irrelevant articles; (2) 53 were literature reviews; (3) 2 were experimental studies; (4) 6 were editorials and letters; (5) 18 were conference abstracts; (6) 6 were guidelines; (7) 22 were books/chapters; (8) 4 were case reports; and (9) 2 were published in other languages. Consequently, a comprehensive evaluation of the full texts of the remaining 25 articles for eligibility assessment was conducted. Of these, 13 articles were excluded due to the following reasons: (1) 2 were case reports [26,27]; (2) 4 were conference abstracts [28,29,30,31]; (3) 3 were letters [32,33,34]; and (4) 4 were literature reviews [13,35,36,37]. The overall number of included articles in the qualitative synthesis was 12 studies, among which 6 were included in the quantitative synthesis [15,17,18,19,38,39,40,41,42,43,44,45].

### 3.2. Study Characteristics

The main characteristics of the included studies are presented in Supplementary Table S1. This systematic review and meta-analysis comprised a total of 883 individuals. The sex distribution was higher for females (472 females (53.45%), 411 males (46.54%)). One study did not specify the sex of the included patients [40]. DD was present in 449 subjects (50.84%) from the total study sample. Among those with colonic diverticula, 5.56% had asymptomatic diverticulosis (AD), 10.46% had SUDD, 4.45% had acute uncomplicated diverticulitis (AUD), 6.23% had acute complicated diverticulitis (ACD), and 8.90% had segmental colitis associated with diverticulosis (SCAD), while in 64.36% of the participants, the type of DD was not specified. Nine studies were carried out in Europe (Italy *n* = 7, United Kingdom *n* = 1, Germany *n* = 1), one in North America (USA *n* = 1), one in Asia (Israel *n* = 1), and one in Russia (*n* = 1).

### 3.3. Definition of Diverticulosis

Colonic diverticula were assessed using colonoscopy for diagnosing DD in most studies (n = 10) [15,18,19,38,39,40,41,45], while two studies used the clinical data reported and used the specimens of the sigmoid colon obtained during sigmoid resection or left hemicolectomy [43,44]. Moreover, two studies used a CT scan for the exclusion of complications in AUD subjects, followed by colonoscopy [17,42].

### 3.4. TNF-α Levels in SUDD Patients vs. Healthy Controls

**Figure 2:**
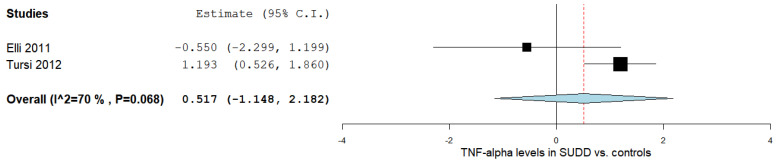
TNF-α levels in SUDD vs. the controls [17,38]. SUDD: symptomatic uncomplicated diverticular disease; TNF-α: tumor necrosis factor-alpha.

Two studies reported the TNF-α levels in SUDD patients and control subjects [17,38]. Figure 2 outlines the obtained meta-analysis results. The pooled analysis evaluating the TNF-α levels in patients with SUDD vs. the control subjects revealed an overall MD of 0.517 (95% CI −1.148–2.182). A substantial level of heterogeneity was reported; I^2^ = 70% and a *p*-value = 0.068.

### 3.5. TNF-α Levels According to DD Status

**Figure 3:**
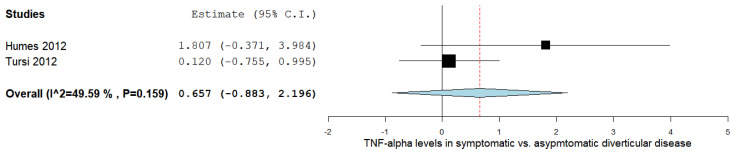
TNF-α levels in symptomatic vs. asymptomatic diverticular disease [17,39]. TNF-α: tumor necrosis factor-alpha.

The TNF-α levels were analyzed in a total of two studies comparing the values in symptomatic with asymptomatic DD patients, as outlined in Figure 3 [17,39]. The pooled analysis assessing the TNF-α levels in symptomatic vs. asymptomatic DD subjects revealed an overall MD of 0.657 (95% CI −0.883–2.196). A moderate level of heterogeneity was reported; I^2^ = 49.59% and a *p*-value = 0.159.

### 3.6. TNF-α Levels in DD vs. IBS

**Figure 4:**
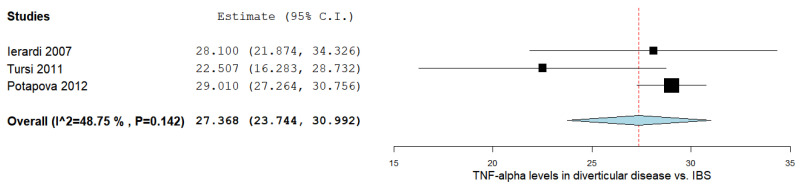
TNF-α levels in diverticular disease vs. IBS [15,37,40]. IBS: irritable bowel syndrome; TNF-α: tumor necrosis factor-alpha.

There was a significant MD between the DD and IBS subjects in the three studies comparing values on the TNF-α levels, as shown in Figure 4 [15,37,40]. The pooled analysis evaluating the TNF-α levels in DD and IBS subjects revealed an overall MD of 27.368 (95% CI 23.744–30.992). A moderate level of heterogeneity was reported; I^2^ = 48.75% and a *p*-value = 0.142.

### 3.7. TNF-α Levels between SCAD and IBS Patients

**Figure 5:**
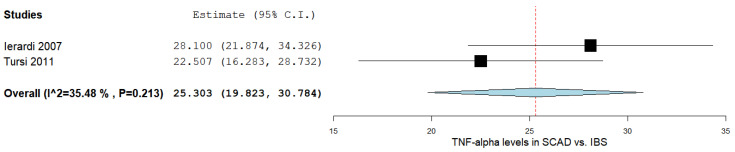
TNF-α levels in SCAD vs. IBS [15,37]. IBS: irritable bowel syndrome; SCAD: segmental colitis associated with diverticulosis; TNF-α: tumor necrosis factor-alpha.

In the two studies comparing the TNF-α levels in SCAD and IBS subjects [15,37], there was a significant MD between the two groups, as outlined in Figure 5. The pooled analysis of the included studies that evaluated the TNF-α levels in SCAD and IBS subjects revealed an overall MD of 25.303 (95% CI 19.823–30.784). A moderate level of heterogeneity was reported; I^2^ = 35.48% and a *p*-value = 0.213.

### 3.8. Qualitative Analysis

A study conducted by Ierardi et al., included 26 subjects, divided into 13 SCAD cases and 13 IBS controls [18]. Over-expression of TNF-α was observed in all SCAD patients, indicating its potential involvement in SCAD pathogenesis. Tursi et al., conducted a study in which a total of 51 participants were included, with 21 SCAD cases (8 type A SCAD, 6 type B SCAD, 3 type C SCAD, and 4 type D SCAD), and 30 serving as controls (10 IBS, 10 moderate-to-severe active UC, and 10 moderate-to-severe active ileo-colonic CD) [15]. The authors reported that TNF-α was significantly over-expressed in all SCAD patients, and higher TNF-α levels were associated with greater endoscopic damage severity. Another study conducted by Tursi et al., involved 20 subjects, including individuals with type B and type D SCAD and those with moderate-to-severe active UC [41]. The results confirmed that TNF-α expression plays a crucial role in the disease activity of SCAD, similar to its involvement in IBD. Furthermore, Tursi et al., carried out an investigation on 24 cases with DD (12 AUD, 12 SUDD) and 30 controls (12 AD, 6 SCAD, 6 UC, 6 HC), in which the authors found that TNF-α expression in DD appeared to be correlated with disease severity [17]. This was also seen in another study conducted by Tursi et al., including 22 cases with DD (15 AUD, 7 SUDD) and 37 controls (13 AD, 10 type B SCAD, 7 UC, 7 HC) [42].

Elli et al., conducted a study including 20 participants, 10 with symptomatic SUDD and 10 healthy controls [38]. The findings demonstrated the absence of inflammatory changes in the colonic mucosa of individuals with symptomatic SUDD. Moreover, Humes et al., evaluated 25 subjects, with 12 having SUDD and 13 serving as controls with asymptomatic DD [39]. The symptomatic patients exhibited a higher median relative expression of TNF alpha mRNA compared to the asymptomatic patients. Potapova et al., performed an investigation on 50 participants, including 25 with DD and 25 controls with IBS [40]. The concentration of TNF-α was significantly higher in DD compared to IBS.

Tursi et al., carried out a study involving 20 cases of ACD and 15 controls [43]. TNF-α was significantly over-expressed in ACD compared to CD, suggesting its involvement in the disease. Peery et al., performed a study on 225 individuals with DD and 364 controls [19]. No evidence was found to support the association of colonic diverticula with mucosal inflammation. Cossais et al., evaluated a total of 39 patients with DD and 23 controls [44]. Although not statistically significant, there appeared to be an increase in TNF-α expression in patients with DD compared to the controls. Moreover, Lahat et al., conducted a study that involved eight patients with ACD and eight patients with AUD [45]. The patients who experienced severe AD had higher tissue inflammatory cytokine levels compared to those with non-severe AD.

### 3.9. Bias Evaluation

The bias risk in individual studies was assessed using the NOS tool, as delineated in Supplementary Table S2. The NOS for cross-sectional studies was used in a total of 12 studies [15,17,18,19,38,39,40,41,42,43,44,45]. Each study had a well-defined research question and objective, while the sample size was small. Only three studies reported consecutive sampling. No study precomputed a sample size. Correcting for confounding factors, by excluding criteria in patient selection, was performed in eight studies. No matching or multiple regression techniques were employed for confounding control. All included studies correctly ascertained the exposure and the outcome. All studies used the correct statistical analysis and reported *p*-values. There were no problems with non-respondents in any of the studies.

## 4. Discussion

Over the past century, the incidence of DD has increased significantly [46,47]. For patients with complicated diverticulitis, several studies have demonstrated varying morbidity and mortality rates. While morbidity could reach 44% [48], mortality rates range from as low as 1% to 16.7% [49]. The pathophysiology of DD is poorly understood, and several etiological factors, such as inflammatory markers, may contribute to the development of the disease. This systematic review and meta-analysis assessed the TNF-α levels in DD compared with IBS, SCAD, and IBS. We also evaluated the relationship between TNF-α levels and DD symptomatic status, and between SUDD and healthy controls. We included 12 articles with a total population of 883 in our qualitative synthesis, out of which 6 studies were included in our quantitative synthesis. We revealed significantly increased colonic mucosal TNF-α levels in DD patients compared to IBS patients, as well as in SCAD patients compared to IBS patients, indicating that TNF-α may be involved in the pathogenesis of this condition. Furthermore, no significantly different TNF-α levels were reported between patients with SUDD and healthy control subjects, and between symptomatic and asymptomatic DD patients. To the best of our knowledge, this is the first systematic review and meta-analysis evaluating this topic.

Diverticulosis is characterized by the presence of pouch-like protrusions (diverticula) that form when the inner layers of the colon wall, including the mucosa and submucosa, bulge through weak areas in the muscular layer [50]. These diverticula are believed to develop due to age-related degeneration of the mucosal wall and localized increases in colonic pressure, resulting in bulging at vulnerable points, particularly where the vasa recta are inserted [50]. The precise underlying mechanisms responsible for the formation of colonic diverticula are not yet fully understood. It is likely that a complex interplay of factors, including age, diet, colonic microbiota, genetic factors, colonic motility, and changes in colonic structure, contribute to this process [51].

In diverticulosis, there should be no evidence of inflammation observed during endoscopic and histological examinations. However, a different scenario may be observed in patients with SUDD. In these patients, low-grade inflammation appears to play a significant role in the occurrence of symptoms and complications [50]. TNF-α, a 17-kDa protein produced by macrophages, lymphocytes, and natural killer cells, was identified as a key player in the inflammatory process, as elevated levels have been found in patients with inflammatory bowel disease (IBD) and rheumatoid arthritis [52,53]. Nevertheless, the role of TNF-α in DD remains a subject of debate, as conflicting results have been reported.

In our meta-analysis, we found that the colonic mucosal TNF-α levels in patients with SUDD in comparison to healthy controls were not significantly different. This could be due to various reasons, including the distinct methods used to detect TNF-α. Moreover, Elli et al., did not include patients with SUDD alone [38], as some of the patients had coexisting IBS, which may be a confounding factor. The limited number of studies included in the analysis may contribute to the high level of heterogeneity and may affect the generalizability of the findings. In contrast, Tursi et al., revealed a significantly higher inflammatory infiltrate in DD in comparison to healthy controls, which was associated with disease severity [54]. They examined DD biopsies using inflammatory count assays or density, while Elli et al. [38] and Tursi et al. [17] used a chemiluminescent multiparametric assay and a reverse transcription polymerase chain reaction (PCR). Lahat et al., revealed that significantly high mucosal TNF-α gene expression levels were observed after severe episodes of acute diverticulitis compared to controls, while an increase in cytokine was not seen after non-severe diverticulitis [45].

Furthermore, we reported that there was no significant difference in the TNF-α levels between symptomatic and asymptomatic subjects with DD. Tursi et al., showed that patients with asymptomatic diverticulosis possessed elevated inflammatory infiltrate in comparison to healthy controls [54]. However, they did not compare asymptomatic and symptomatic DD patients. Our findings suggest that TNF-α levels are not directly related to the presence or absence of symptoms in DD patients.

We reported significantly increased TNF-α levels in DD patients compared to IBS patients, as well as in SCAD patients compared to IBS patients. SCAD showed the highest TNF-α expression, which strongly indicates that TNF-α expression in DD is related to the severity of the inflammatory process. These results are in accordance with previous studies that showed that the TNF-α expression in SCAD and UC was similar [55]. These observations suggest that SCAD pathogenesis may mirror the pathogenic models proposed for IBD.

Our reported findings may have an application in clinical practice. First, TNF-α may be a useful biomarker for distinguishing between DD and IBS, which can have similar clinical presentations. IBS is not associated with the over-expression of TNF-α [18,56]. The association between TNF-α and DD was previously suggested in several studies, and our analysis also revealed a significant difference in the TNF-α levels between patients with DD and those with IBS, as well as between patients with SCAD and those with IBS. However, it is worth noting that the level of heterogeneity in these analyses was lower than in the analysis of SUDD vs. healthy control subjects, suggesting that the results may be more reliable. Second, the over-expression of TNF-α provides a potential therapeutic target for the management of DD, especially SCAD. Anti-inflammatory drugs, such as 5-aminosalicylic acid (5-ASA) or anti-TNF-α agents, such as infliximab and adalimumab, have been used in the treatment of other inflammatory conditions, such as inflammatory bowel disease (IBD). Understanding the role of TNF-α over-expression in DD may pave the way for the development of targeted therapies, aimed at modulating the inflammatory response and improving patient outcomes. Further studies are warranted to explore whether anti-inflammatory drugs can result in clinical improvement that correlates with the downregulation of TNF-α.

The meta-analysis results should be interpreted with the following limitations taken into consideration. First, the number of included studies was relatively small, which limits the generalizability of our findings. Second, some studies had limitations in terms of the representativeness of the sample, as they included only specific populations, such as patients referred for colonoscopy or those undergoing surgery. Third, some studies did not report or adequately control for potential confounding factors, such as age, sex, BMI, and comorbidities, which may have influenced the results. Fourth, there was significant heterogeneity among the included studies, which could also have contributed to the risk of bias. Fifth, causality cannot be confirmed nor negated in our evaluated assessments due to the cross-sectional design of the included studies. Sixth, further subgroup analyses according to DD subtypes were not possible due to scarce publications on this topic. Despite these limitations, our study provides valuable insights into the potential role of TNF-α in the pathogenesis of DD. Future research with larger sample sizes and more rigorous methodologies is needed to elucidate the exact role of TNF-α in the pathogenesis of this condition, to determine its potential as a biomarker for diagnosis, and to explore the potential therapeutic implications of targeting TNF-α in the management of DD.

Nevertheless, this systematic review and meta-analysis have several strengths. We utilized a comprehensive search strategy using multiple electronic databases. Moreover, this is the first systematic review and meta-analysis on this topic, providing a valuable contribution to the existing literature, identifying current gaps in evidence regarding TNF-alpha in DD, and serving as a starting point for future research.

## 5. Conclusions

No significant differences in the colonic mucosal TNF-α levels between SUDD vs. the controls, and symptomatic vs. asymptomatic DD were found. However, the TNF-α levels were significantly increased in DD patients compared to IBS patients, as well as SCAD patients compared to IBS patients.

## Data Availability

The analyzed data was extracted from the cited original articles; the quality assessment data is published in the Supplementary File. Further enquiries can be directed to the corresponding author.

## Supplementary Materials

The following supporting information can be downloaded at: https://www.mdpi.com/article/10.3390/ijms24129934/s1.
