# Supplementary material for: Do Colonic Mucosal Tumor Necrosis Factor Alpha Levels Play a Role in Diverticular Disease? A Systematic Review and Meta-Analysis
Source: Int J Mol Sci. 2023 Jun 9;24(12):9934. doi: 10.3390/ijms24129934 (PMC10298590; doi:10.3390/ijms24129934)
Supplement: Supplementary file 1 [file ijms-24-09934-s001.zip › ijms-2428340-supplementary.pdf]

Supplementary Table S1. Studies evaluating TNF-α levels in diverticular disease

First Author / Year / Country	Study Design	Study Characteristics	Main Findings
**Ierardi et al. / 2008 / Italy**	Cross-sectional	• **Population:** SCAD and IBS controls<br>• **Total Subjects:** 26 (Cases: n = 13, [SCAD = 13]); (Controls (IBS): n =13)<br>• **Diverticular disease patients:** 13 (50 %)<br>• **Mean age (years):** (SCAD: 66.5 years, age range 49–80 years); Controls (IBS): 65.6 years; range 47–78 years<br>• **Sex (males):** SCAD 8 (61.5%); Control (IBS) 8 (61.5%)<br>• **TNF-α Measurement Method:** immunohistochemistry<br>• **TNF-α level (ng/ml)**: SCAD (*n*=13): 38.6 ± 10.4%; Controls (*n*=13): 10.5 ± 4.8% (P<0.001)<br>• **TNF-α measurement**: biopsy specimens	Over-expression of TNF-α was found in all SCAD patients. The data suggest that TNF-α activity is involved in SCAD pathogenesis.
**Tursi et al. / 2010 / Italy**	Cross-sectional	• **Population:** SCAD (type A, type B, type C, type D) and Control groups (IBS, moderate-to-severe active UC, moderate-to-severe active ileo-colonic CD)<br>• **Total Subjects:** 51, (SCAD: 21 [type A SCAD = 8, type B SCAD = 6, type C SCAD = 3, type D SCAD = 4]); (Controls: n = 30 [IBS = 10, moderate-to-severe active UC = 10, moderate-to-severe active ileo-colonic CD = 10)<br>• **Diverticular disease patients:** 21 (41.1%)<br>• **Mean age (years):** SCAD: 58.87 years, range 43–85 years; IBS: 56.57 years, range 27–68 years; UC: 38.27 years, range 23–48 years; CD: 35.47 years, range 21–45 years<br>• **Sex (males):** SCAD 15 (71.4%); Control 13 (43.3%)<br>• **TNF-α Measurement Method:** immunohistochemistry<br>• **TNF-α level (pg/ml)**:<br>- type A SCAD (n = 8): 19.1 (2.54; 95% C.I. 0.18–0.21);<br>- type B SCAD (n = 6): 42.7 (7.58; 95% C.I. 0.24–0.63);<br>- type C SCAD (n= 3): 21.1 (2.6, 95% C.I. 0.16–0.28);<br>- type D SCAD (n = 4): 40 (5.9, 95% C.I. 0.27–0.65);<br>- IBS (n = 10): 8 (4, 95% C.I. 0.2–0.16);<br>- moderate-to-severe active UC (n = 10): 45.5 (5.9, 95% C.I. 0.27–0.65)<br>- moderate-to-severe active ileo-colonic CD (n = 10): 38.1 (3.9, 95% C.I. 0.22–0.55)<br>• **TNF-α measurement**: biopsy specimens	The results of our study show that a high expression of TNF-α is also present in patients with SCAD. In addition, not only a TNF-α over-expression was present without exception in all the SCAD patients enrolled in our study, but the mean levels of TNF-α seem to be related to the severity of the endoscopic damage.
**Tursi et al. / 2011 / Italy**	Cross-sectional	• **Population:** SCAD (type B, type D) and moderate-to-severe active UC<br>• **Total Subjects:** 20 (Cases: n =10, [type B SCAD = 6, type D SCAD = 4]); (Controls: n = 10)<br>• **Diverticular disease patients:** 10 (50%)<br>• **Mean age (years):** SCAD: 58.87 years, range 43-85 years; Controls: 38.27 years, range 23-48 years	This study confirms that TNF-α expression plays a crucial role in the disease activity of SCAD, resembling to what occurs in IBD.

		• **Sex (males):** SCAD: 6 (60 %); Control: 6 (60 %)<br>• **TNF-α Measurement Method:** immunohistochemistry<br>• **TNF-α level (pg/ml)**:<br>- type B SCAD (n = 6): 42.7 (±7.58; 95% C.I. 0.24–0.63);<br>- type D SCAD (n = 4): 40 (±5.9, 95% C.I. 0.27–0.65);<br>- moderate-to-severe active UC (n = 10): 45.5 (±5.9, 95% C.I. 0.27–0.65)<br>**TNF-α measurement**: biopsy specimens	
**Elli et al. / 2011 / Italy**	Cross-sectional	• **Population:** SUDD and HC<br>• **Total Subjects:** 20 (Cases: n = 10, [SUDD = 10]); (Controls: n = 10)<br>• **Diverticular disease patients:** 10 (50%)<br>• **Mean age (years):** SUDD: 66 ± 10 years; range, 41–79 years; Controls: 60 ± 9 years; range, 42–77 years<br>• **Sex (males):** SUDD 5 (50%); Control 5 (50%)<br>• **TNF-α Measurement Method:** chemoluminescent multiparametric assay (Endogen, Bologna, Italy)<br>• **TNF-α level (pg/ml)**: SUDD (n=10): 2.03 ± 1.98, HC (n=10): 2.58 ± 2.01, p: NS<br>• **TNF-α measurement**: biopsy specimens	The present study demonstrates the absence of an inflammatory alteration in the colonic mucosa of patients affected by symptomatic UDD.
**Humes et al. / 2012 / UK**	Cross-sectional	• **Population:** SUDD and AD<br>• **Total Subjects:** (Cases: n =25, [SUDD = 12]); (Controls: n =13)<br>• **Diverticular disease patients:** 12 (48 %)<br>• **Mean age (years):** SUDD: 66.5 years, range 49–71 years; Controls: 63 years, range: 59.5–68.0 years<br>• **Sex (males):** SUDD: 9 (75%); Control: 6 (46.1%)<br>• **TNF-α Measurement Method:** Real time PCR<br>• **TNF-α level (pg/ml)**: SUDD (n=12): 3.17; i.q.r 2.10–6.25; AD (n= 13): 2.16; i.q.r 0.94–3.00, p: 0.04<br>• **TNF-α measurement**: biopsy specimens	Symptomatic patients had greater median relative expression of TNF-α mRNA compared with asymptomatic patients.
**Potapova et al. / 2012 / Russia**	Cross-sectional	• **Population:** DD and IBS<br>• **Total Subjects:** Cases: n =50, ([DD=25]); (Controls: n=25)<br>• **Diverticular disease patients:** 25 (50%)<br>• **Mean age (years):** SUDD: 57.6±9.4 years<br>• **Sex (males):** Not specified<br>• **TNF-α Measurement Method:** ELISA (Cytokine and Protein Contour commercial kits)<br>• **TNF-α level (pg/g)**: DD (n=25): 299.3±34.5; Controls (n=25): 9.2±0.8, p <0.05<br>• **TNF-α measurement**: biopsy specimens	TNF-α concentration in DD was significantly higher than in IBS.

**Tursi et al. / 2012 / Italy**	Cross-sectional	• **Population:** DD (AUD, SUDD) AND Controls (AD, type B SCAD, UC, HC)<br>• **Total Subjects:** (Cases: n= 24, [AUD = 12, SUDD = 12]); (Controls: n = 30 [AD=12, SCAD=6, UC=6, HC=6)<br>• **Diverticular disease patients:** 42 (77,7%)<br>• **Mean age (years):** DD: 67.5 years, interquartile range 15.5; Controls: AD: 71.5 years, IQR 11.0; type B SCAD: 66.5 years, IQR 12; UC: 66.0 years, IQR 9.8; HC: 70.5 years, IQR 28.8<br>• **Sex (males):** DD: 9 (37.5%); Control: 11 (36.6%)<br>• **TNF-α Measurement Method:** RT-PCR<br>• **TNF-α level (pg/ml)**:<br>AUD (n=12): 2.98 (1.92–8.63)<br>SUDD (n=12): 2.1 (1.7–2.9)<br>AD (n=12): 1.5 (1.04–1.79)<br>type B SCAD (n=6): 3.9 (3.1–3.9)<br>UC (n=6): 4.4 (3.1–6.63)<br>HC (n=6): 1 (1–1.10)<br>• **TNF-α measurement**: biopsy specimens	TNF-α expression in DD seems to be related to the severity of the disease.
**Tursi et al. / 2012 / Italy**	Cross-sectional	• **Population:** DD (AUD, SUDD) and Controls (AD, type B SCAD, UC, HC)<br>• **Total Subjects:** (Cases: n =22, [AUD = 15, SUDD = 7]); (Controls: n = 37 [AD=13, type B SCAD=10, UC=7, HC=7)<br>• **Diverticular disease patients:** 45 (76.27%)<br>• **Mean age (years):** DD: 66.5, range 31–79; Controls: AD: 71.5 years, range 45–89; type B SCAD: 66.5 years, range 48–86; UC: 66.0 years, range 40–79; HC: 70.5 years, range 41–83,<br>• **Sex (males):** DD: 8 (36.6%); Control: 22 (59.4%)<br>• **TNF-α Measurement Method:** RT-PCR<br>• **TNF-α level (pg/ml)**:<br>AUD (n= 15): 2.11 (1.73–3.09)<br>SUDD (n= 7): 2.06 (1.76–2.74)<br>AD (n= 13): 1.49 (1.49–1.51)<br>type B SCAD (n= 10): 4.14 (4.12–4.17)<br>UC (n= 7): 1.72 (1.71–1.83)<br>HC (n= 7): 0.99 (0.99–1.00)<br>• **TNF-α measurement**: biopsy specimens	TNF-α expression in DD seems to be related to the severity of the disease.
**Tursi et al. / 2014 / Italy**	Cross-sectional	• **Population:** ACD and stricturing CD<br>• **Total Subjects:** ACD: n = 20; Controls: n = 15<br>• **Diverticular disease patients:** 20 (57.14%)<br>• **Mean age (years):** ACD: 68.5 years, range 41–81 years; Controls: 38.5 years, range 21–51 years<br>• **Sex (males):** 12 (60%); Control 6 (40 %)<br>• **TNF-α Measurement Method:** RT-PCR<br>• **TNF-α level (pg/ml)**: described in figure without reported values	TNF-α are significantly overexpressed in ACD with respect to CD.

		• **TNF-α measurement**: biopsy specimens	
**Peery et al. / 2018 / SUA**	Cross-sectional	• **Population:** DD and Controls<br>• **Total Subjects:** DD = 225; Controls: n = 364<br>• **Diverticular disease patients:** 225 (38.20%)<br>• **Mean age (years):** Not reported<br>• **Sex (males):** DD: 120 (47%); Controls: 150 (41.2%)<br>• **TNF-α Measurement Method:** RT-PCR<br>• **TNF-α level (pg/ml)**: Diverticulosis and TNF-α: OR 0.85; 95% CI, 0.63–1.16)<br>• **TNF-α measurement**: biopsy specimens	No evidence that colonic diverticula are associated with mucosal inflammation.
**Cossais et al. / 2018 / Germany**	Cross-sectional	• **Population:** DD and nonobstructive colorectal carcinoma<br>• **Total Subjects:** DD: n = 39; Controls: n = 23<br>• **Diverticular disease patients:** 39 (62.9%)<br>• **Mean age (years):** DD: 61 years; Controls: 69.3 years<br>• **Sex (males):** DD: 14 (35.8%); Controls: 13 (56.5%)<br>• **TNF-α Measurement Method:** RT-PCR<br>• **TNF-α level (pg/ml)**: described in figure without reported values<br>• **TNF-α measurement**: biopsy specimens	TNF-α expression appeared to be increased in patients with DD in comparison with controls, although this difference was not significant.
**Lahat et al. / 2018 / Israel**	Cross-sectional	• **Population:** after suffering an episode of ACD and after suffering an episode of AUD<br>• **Total Subjects:** ACD: n = 8; AUD: n=8<br>• **Diverticular disease patients:** 16 (100%)<br>• **Mean age (years):** ACD: 55.7±14.4 years, range 36-77; AUD: 62.4±4.5 years, range 58-72<br>• **Sex (males):** ACD: 4 (50%); AUD: 3 (60%)<br>• **TNF-α Measurement Method:** RT-PCR<br>• **TNF-α level (pg/ml)**: severe AD (n=8): 5.4±4.4, non-severe AD (n=8): 0.19±0.11<br>• **TNF-α measurement**: biopsy specimens	Patients after severe AD have higher tissue inflammatory cytokine levels than patients after nonsevere AD.
**DD-** diverticular disease**; AD -** asymptomatic diverticulosis; **SUDD -** symptomatic uncomplicated diverticular disease; **SCAD -** colitis associated with diverticula; **AUD** - acute uncomplicated diverticulitis; **ACD -** acute complicated diverticulitis; **IBS** - irritable bowel syndrome; **UC** - ulcerative colitis; **CD** - Crohn's disease; **HC** - Healthy Controls; **ELISA** – Enzyme-linked immunosorbent assay; **PCR** - Reverse transcription polymerase chain reaction **; IQR** - interquartile range; NS - Not significant;			

Supplementary Table S2. The Newcastle-Ottawa Scale (NOS) for assessing the quality of cross-sectional studies

Study	Selection				Comparability	Outcome		Score
	Sample representativeness	Sample size	Non-Respondents	Ascertainment of the exposure (risk factor)	Comparability	Assessment of the outcome	Statistical test	
Ierardi et al. 2008	*	-	*	*	*	*	*	6
Tursi et al. 2010	-	-	*	*	*	*	*	5
Tursi et al. 2011	-	-	*	*	*	*	*	5
Elli et al. 2011	-	-	*	*	*	*	*	5
Humes et al. 2012	*	-	*	*	-	*	*	5
Potapova et al. 2012	-	-	*	*	-	*	*	4
Tursi et al. 2012	-	-	*	*	*	*	*	5
Tursi et al. 2012	-	-	*	*	*	*	*	5
Tursi et al. 2014	-	-	*	*	-	*	*	4
Peery et al. 2018	-	-	*	*	*	*	*	5
Cossais et al. 2018	-	-	*	*	-	*	*	4
Lahat et al. 2018	*	-	*	*	*	*	*	6
